# Association of Platelet Binding to Lymphocytes with B Cell Abnormalities and Clinical Manifestations in Systemic Lupus Erythematosus

**DOI:** 10.1155/2019/2473164

**Published:** 2019-03-03

**Authors:** Carlos Zamora, Elide Toniolo, Cesar Diaz-Torné, Elisabet Cantó, Berta Magallares, Ma Angels Ortiz, Lidia Perea, Hector Corominas, Silvia Vidal

**Affiliations:** ^1^Department of Immunology, Biomedical Research Institute Sant Pau (IBB Sant Pau), Barcelona, Spain; ^2^Unit of Rheumatology, Hospital de la Santa Creu i Sant Pau, Barcelona, Spain

## Abstract

Systemic lupus erythematosus (SLE) is an autoimmune disease associated with the polyclonal activation of B lymphocytes and the production of autoantibodies that cause immune complex-related inflammation. Immunological factors derived from platelets modulate B cell function in SLE disease. However, platelets do not only modify the immune system by soluble factors. The binding of platelets to lymphocytes can modulate immune response. Thus, we speculate that the binding of platelets to lymphocytes in SLE patients may play a role in abnormal B lymphocyte response and the pathogenesis of SLE. We observed that levels of lymphocytes with bound platelets were higher in SLE patients than in healthy donors (HD). In SLE patients, the percentage of B lymphocytes with bound platelets positively correlated with plasmatic levels of IgG, IgA, IL-10, and soluble CD40L and negatively correlated with IgM levels, though not in HD. Preswitched memory B lymphocytes were the subpopulation with more bound platelets. Lymphocytes with bound platelets from both HD and SLE patients had major levels of CD86 and BAFFR and a greater production of IL-10 than lymphocytes without bound platelets. However, only B lymphocytes with bound platelets from SLE patients had increased levels of IgG and IgA on their surface. SLE patients with a suggestive renal manifestation had the highest levels of B and T lymphocytes with bound platelets. These results suggest that the binding of platelets to lymphocytes plays a role in SLE disease and that controlling this binding may be a promising therapeutic approach.

## 1. Introduction

SLE has been associated with polyclonal B cell hyperactivity and autoantibody production [1–3]. B lymphocytes from SLE patients also display an increased expression of the activated markers CD86, CD80, and CD38 [4, 5] and IgG and IgA [6] levels on their surface. These patients have an elevated proportion of memory B lymphocytes and a decreased proportion of preswitched B lymphocytes [7, 8].

IL-10 is an anti-inflammatory cytokine secreted by Tregs, Bregs, Th2 lymphocytes, monocytes, and other cells that plays a crucial role in preventing inflammatory and autoimmune disease [9]. However, there is an elevated concentration of plasma IL-10 in SLE and these levels correlate with disease activity (SLEDAI) and the production of anti-dsDNA antibodies [10]. IL-10 has a positive influence on B cell survival, proliferation, differentiation, and autoantibody production [9]. Blocking IL-10 in SLE patients decreases autoantibody production and reduces clinical symptoms and disease activity [11, 12]. This IL-10 overproduction in SLE patients has been ascribed to B lymphocytes and monocytes [13]. Since B regulatory lymphocytes are the major B lymphocytes producing IL-10, they likely contribute to SLE pathogenesis [14, 15].

Platelet has recently been recognized as an immunoregulatory cellular component [16]. Upon activation, platelets release cytokines, chemokines, growth factors, and platelet-derived microparticles (PMP) and express a set of activation molecules on their membrane (P-selectin and CD40L) that allow the platelet binding to leukocytes [17–20]. Platelets can bind to lymphocytes and P-selectin-PSGL-1 appears to be essential in this interaction. However, other molecules, such as CD40-CD40L, GPIb-CD11b, and GPIIb/IIIa-CD11/CD18, can also play a role in the binding of platelet to lymphocytes [21]. Platelets directly modulate humoral activity, stimulating B cell proliferation, antibody production, and the membrane expression of CD27 and CD86, via soluble CD40L (sCD40L) [22, 23]. Both mechanisms, platelet-derived soluble factors (TGF*β*, PF4, and sCD40L) and the binding of platelets to leukocytes (CD62P-PSGL1), decrease T cell proliferation and inflammatory cytokine production and increase IL-10 production by T lymphocytes and monocytes [18–20, 24, 25]. Through CD40-CD40L ligation, platelets support B cell isotype switching [23, 26] and, with the collaboration of Th cells, platelets enhance germinal center formation [27]. In fact, CD40L-deficient mice failed to elicit B cell isotype switch and this failure could be corrected by infusing CD40L-expressing platelets or platelet-free supernatant from activated platelets containing sCD40L [26]. Similarly, the blocking of CD40L in cocultures of platelets with B lymphocytes partially abolishes the binding of platelets to B lymphocytes [23]. In SLE patients, high levels of plasma sCD40L are observed, which are associated with disease severity and the development of nephritic lupus [28, 29]. sCD40L is released from platelet surface after its activation, and it is considered an in vivo marker of platelet activation [30, 31]. However, since CD40L is overexpressed on SLE T lymphocytes [28, 32, 33], and it can be shed from the membrane [34]; plasmatic sCD40L in SLE patients is not the sole product of platelets. An elevated number of circulating lymphocyte-platelet complexes and an increased platelet activation have been reported in patients with SLE and other autoimmune diseases [19, 35]. However, the binding of platelets to different lymphocyte subpopulations in SLE patients has not yet been studied and there is a lack of information about the relationship between platelet-binding to lymphocytes, lymphocyte function, and the clinical outcome of these patients.

Our aim was to analyze the possible role of lymphocyte-platelet complexes in the altered B cell function and SLE pathogenesis. Firstly, we compared the numbers of lymphocytes with bound platelets in SLE patients and healthy donors. Secondly, we established their relationship with platelet activation and the isotype class of autoantibodies and we investigated the phenotype and function of B lymphocytes with bound platelets in SLE patients. Finally, we established the relationship between lymphocytes with bound platelets and the clinical (hematuria and SLEDAI) and laboratory (anti-dsDNA, complement C3, and albumin/creatinine) features of patients.

## 2. Material and Methods

### 2.1. Study Subjects and Sample Collection

Whole blood from 16 healthy donors (HD) and 21 SLE patients was collected in BD vacutainer tubes containing heparin (BD, Franklin Lakes, NJ). SLE diagnosis was based on 1982 revised ACR criteria [36]. Two of the 21 patients did not meet to the Systemic Lupus International Collaborating Clinics (SLICC) criteria for SLE classification. Patients previously treated with B cell-targeted therapies, with other biologic agents, or with more than 10 mg per day of prednisone were excluded. To assess disease activity, patients' Systemic Lupus Erythematosus Disease Activity Index (SLEDAI) was calculated at the time of the sample collection [37]. In Table 1, we show the demographic, clinical, and laboratory data of SLE patients enrolled in this study. Written informed consent was obtained, and ethical approval for the study was granted by the Hospital de la Santa Creu i Sant Pau Institutional Ethics Committee.

Peripheral blood mononuclear cells (PBMCs) were isolated by Ficoll-Hypaque density gradient (Lymphoprep, AXIS-SHIELD PoCAs, Oslo, Norway).

### 2.2. Staining of Peripheral Blood Mononuclear Cells and Whole Blood Cells

PBMCs (1 × 10^6^ cells) were incubated with anti-CD19-PEDy647, anti-CD38-PE, anti-CD41a-FITC, anti-CD41a-PE (Immunotools, Friesoythe, Germany), anti-CD19-PECy7, anti-CD27-APC, anti-BAFFR-FITC, anti-TACI-PE (BioLegend, San Diego, USA), anti-CD86-PE, anti-IgG-PE, anti-IgD-FITC (BD), anti-IgA-FITC, and anti-IgM-PE (Dako, CA, USA) mAbs and the corresponding isotype controls. Whole blood (100 *μ*l) was incubated with anti-CD4-PECy7 (BioLegend), CD5-PE (BD Biosciences), CD19-PEDy647, and CD41a-FITC (Immunotools). Red blood cells were lysed, and white cells fixed using BD FACS lysing solution (BD Biosciences) to be analyzed by flow cytometry.

### 2.3. Flow Cytometry Analysis of Surface Markers and IL-10 Production

We were able to identify the main T and B lymphocyte subsets combining anti-CD19, anti-CD4, anti-CD8, and anti-CD5. Two subsets of B lymphocytes with bound platelets (PLTs) were identified as CD19+CD5-CD41a+ (CD19+PLT+) and CD19+CD5+CD41a+, respectively. CD4+ and CD8+ T lymphocytes with bound platelets were identified as CD19-CD4+CD5+CD41a+ (CD4+PLT+) and CD19-CD4-CD5+CD41a+ (CD8+PLT+), respectively, on gated lymphocytes. Activation markers and IL-10 production were analyzed on CD19+PLT+ and CD19+PLT- (B lymphocytes without bound platelets). CD19+ and CD19- lymphocytes that secreted spontaneously IL-10 were determined using IL-10 secretion assay (Miltenyi Biotec, Bergisch Gladbach, Germany), a kit that measures IL-10 secretion on the cell surface. Briefly, after 4 h of resting, PBMCs were labeled with IL-10-specific catch reagent on their surface and incubated for 45 min at 37°C under slow rotation. Cells were washed and labeled with IL-10-PE detection antibody, CD19-PECy7 (BioLegend), CD41a-FITC (Immunotools), and viability marker LIVE/DEAD fixable Violet Dead Cell Stain kit (Thermo Fisher Scientific, Waltham, Massachusetts, USA). Then cells were washed, and membrane IL-10 was analyzed by flow cytometry. Percentages of viable lymphocytes did not differ between HD and SLE patients (90-95%). The percentage of CD19+ B lymphocyte subpopulations with or without bound platelets were defined as follows: naïve B lymphocytes (CD27-IgD+CD41+ and CD27-IgD+CD41-, respectively), preswitched memory B lymphocytes (CD27+IgD+CD41a+ and CD27+IgD+CD41a-, respectively), postswitched memory B lymphocytes (CD27+IgD-CD41a+ and CD27+IgD-CD41a-, respectively), and double-negative (DN) memory B lymphocytes (CD27-IgD-CD41a+ and CD27-IgD-CD41a-, respectively) [38]. Samples were acquired with the MACSQuant Analyzer 10 flow cytometer (Miltenyi Biotec), and we determined the percentages of cells and the geometric mean fluorescence intensity (MFI) of BAFFR.

### 2.4. Quantification of Anti-dsDNA, Complement C3, Albumin/Creatinine, Hematuria, IgG, IgM, and IgA

Anti-dsDNA levels were determined in serum using Unicap Elia (Phadia Laboratory Systems, Uppsala, Sweden). Levels of complement C3 in serum and plasma IgG, IgM, and IgA were determined using the Nephelometry System (Beckman Coulter). Albumin/creatinine was determined in serum by the turbidimetry system (Abbott, Chicago, USA). The presence of hematuria was determined by the Combur test (Roche, Basilea, Switzerland). Anti-dsDNA antibodies were considered positive when levels were >17 UI/ml. Levels of albumin/creatinine were expressed as mg/mmol. Levels of complement C3 and immunoglobulins were expressed as mg/dl.

### 2.5. Determination of IL-10 and sCD40L

Plasma concentrations of IL-10 (Immunotools) and sCD40L (Peprotech, London, UK) were determined using specific ELISA kits according to the manufacturers' instructions and using the specific standard curves of recombinant molecules. The limits of detection were as follows: 16 pg/ml for IL-10 and 31.25 pg/ml for sCD40L.

### 2.6. Statistics

The Kolmogorov-Smirnov test was applied to test the data for normal distribution. Normally distributed variables were then reported as mean ± s.e.m. Comparisons between groups were tested with Student's *t*-test, the Mann-Whitney test, or the Wilcoxon test according to Gaussian distribution. ANOVA was used to compare more than two groups with normal distributions. Correlation analyses were carried out with Pearson's correlation. *p* values *<* 0.05 were considered significant.

## 3. Results

### 3.1. Circulating Lymphocytes with Bound Platelets in SLE Patients

**Figure 1:**
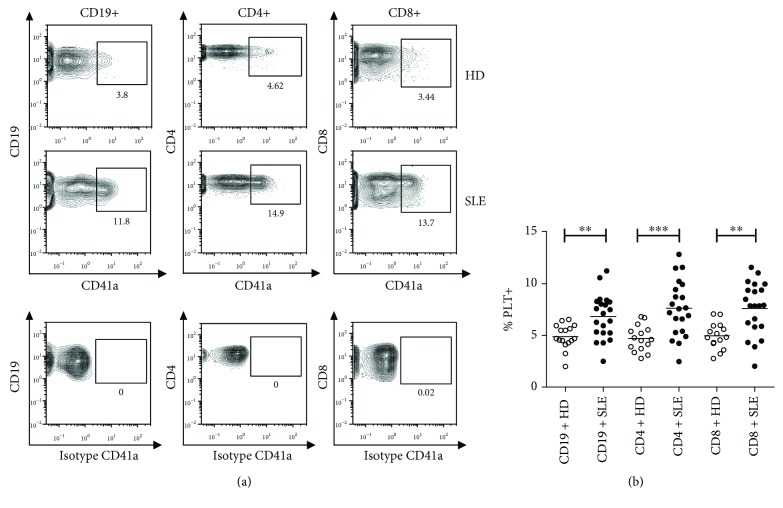
Percentage of B and T lymphocytes with bound platelets in HD and SLE patients. (a) A representative image of B lymphocytes (CD19+), CD4+ T lymphocytes (CD19-, CD4+, and CD5+) and CD8+ T lymphocytes (CD19-, CD4-, and CD5+) with bound platelets (PLTs) in HD and SLE patients is shown. (b) The dot plot shows the percentage of B and CD4 and CD8 T lymphocytes with bound platelets in HD (*n* = 16) and SLE patients (*n* = 21). CD4 and CD8 T lymphocytes had more bound platelets than B lymphocytes in SLE patients (both *p* < 0.01), but not in HD. Data are presented as the mean. Statistical analysis was performed using the *t*-test for comparison between HD and SLE and paired *t*-test for the subpopulation comparisons in HD and SLE. ^∗∗^*p* < 0.01 and ^∗∗∗^*p* < 0.0001.

Higher percentages of circulating B and T lymphocytes with bound platelets were observed in SLE patients than in HD (Figures 1(a) and 1(b)). The percentages of bound platelets to the CD19+CD5+ B cell subpopulation in HD and SLE patients were comparable (7.15 ± 1.04 and 9.98 ± 1.54, respectively). In SLE patients but not in HD, T lymphocytes had more bound platelets than B lymphocytes (*p* < 0.01 for both CD4+ and CD8+ T lymphocytes). The percentages of B, CD4+, and CD8+ T lymphocytes with bound platelets correlated strongly to each other (*r* = 0.92, *p* < 0.0001 for %CD19+PLT+ vs. CD4+PLT+; *r* = 0.87, *p* < 0.0001 for %CD19+PLT+ vs. CD8+PLT+; and *r* = 0.91, *p* < 0.0001 for %CD4+PLT+ vs. CD8+PLT+). The percentage of B and T lymphocytes with bound platelets was not affected by the absolute numbers of lymphocytes and platelets (data not shown).

### 3.2. Association of B Lymphocytes with Bound Platelets with the Levels of Immunoglobulin and Platelet Activation

**Figure 2:**
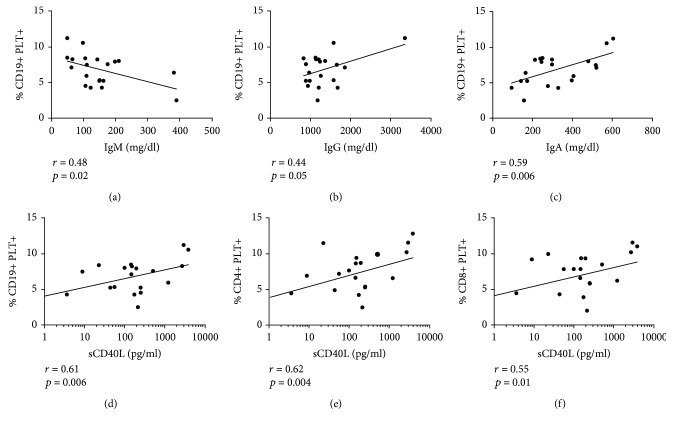
Association of lymphocytes with bound platelets with immunoglobulin's isotypes and platelet activation in SLE patients. Correlation of CD19+PLT+ percentages with levels (mg/dl) of (a) IgM (b) IgG and (c) IgA in 20 SLE patients. Measurement of Igs in one SLE patient could not be performed due to the lack of sample. Correlation of sCD40L levels (pg/ml) with percentage of (d) CD19+PLT+, (e) CD4+PLT+, and (f) CD8+PLT+ in 19 SLE patients. Measurements of sCD40L in two SLE patients could not be performed by the lack of samples. Pearson's correlation was performed for the correlation analysis.

The percentage of CD19+PLT+ correlated positively with concentrations of IgG and IgA levels and correlated negatively with concentrations of IgM in SLE patients (Figures 2(a)–2(c)), though not with HD (*r* = 0.22, *p* = 0.52 for IgM; *r* = 0.08, *p* = 0.81 for IgA; and *r* = 0.31, *p* = 0.39 for IgG). A positive correlation between the percentage of CD19+PLT+ and the IgG/IgM ratio was found in SLE patients (*r* = 0.56, *p* = 0.01) but not in HD. No differences in the plasma concentrations (mg/dl) of IgG, IgA, and IgM were observed between HD and SLE patients (IgG: 1083 ± 34.74 for HD vs. 1348 ± 125 for SLE; IgA: 262.2 ± 42.85 for HD vs. 319 ± 34.39 for SLE; IgM: 129.9 ± 16.55 for HD vs. 149.6 ± 20.76 for SLE). Plasma levels of sCD40L in SLE patients were higher than in HD (50.58 ± 25.79 pg/ml for HD vs. 691.3 ± 268.7 pg/ml for SLE, *p* < 0.01) and correlated with the percentage of each subpopulation of lymphocytes with bound platelets in SLE patients (Figures 2(d)–2(f)), though not in HD (*r* = 0.18, *p* = 0.66 for CD19+PLT+; *r* = 0.5, *p* = 0.25 for CD4+PLT+; and *r* = 0.28, *p* = 0.49 for CD8+PLT+). sCD40L also correlated with IgA levels in SLE patients (*r* = 0.52, *p* = 0.02).

### 3.3. Different Binding of Platelet to Preswitched Memory B Lymphocytes in SLE Patients

**Figure 3:**
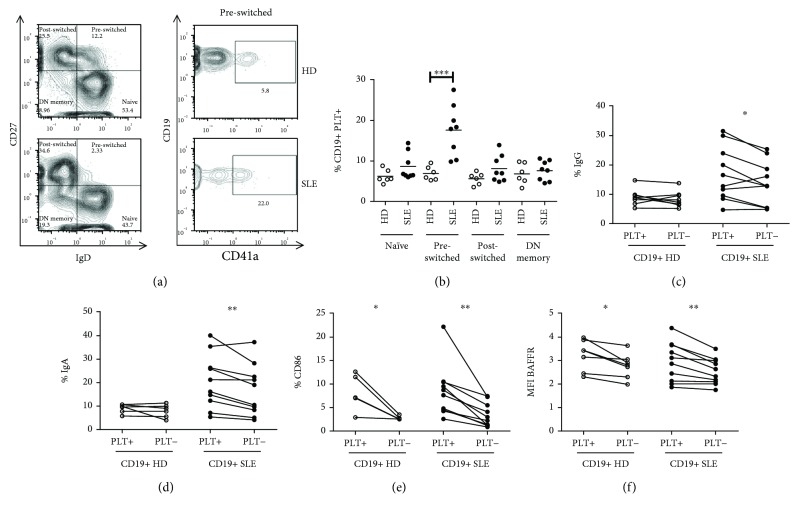
Phenotype and subpopulations of B lymphocytes with bound platelets. (a) A representative image of naïve, preswitched, postswitched, and a double-negative (DN) memory and preswitched memory B lymphocytes with bound platelets from HD and SLE patients. (b) Percentages of CD19+PLT+ in each B lymphocyte subpopulation in HD (*n* = 6) and SLE (*n* = 8). Multiple comparisons were analyzed by one-way ANOVA (*p* < 0.0001 in SLE patients). Percentage of (c) IgG, (d) IgA, (e) CD86, and (f) BAFFR MFI on CD19+PLT+/PLT- from HD (*n* = 6) and SLE (*n* = 10). Data are presented the as mean. Statistical analysis was performed using the Mann-Whitney test for unpaired samples and Wilcoxon test for paired samples. ^∗^*p* < 0.05, ^∗∗^*p* < 0.01, and ^∗∗∗^*p* < 0.001.

As expected [7, 8], SLE patients had a higher percentage of DN memory B lymphocytes but a lower percentage of preswitched memory B lymphocytes than HD (DN memory: 22.62 ± 2.52% for SLE vs. 12.76 ± 2.26% for HD, *p* < 0.05; preswitched memory: 3.31 ± 0.73% for SLE vs. 10.85 ± 1.91% for HD, *p* < 0.01). The percentages of naïve and postswitched memory B lymphocytes were comparable in SLE patients and HD. In the latter group, the different B lymphocyte subpopulations had a similar percentage of cells with bound platelets. However, in SLE patients, preswitched memory B lymphocytes showed a higher percentage of CD19+PLT+ lymphocytes compared with the other B lymphocytes subpopulations (*p* < 0.0001 by ANOVA) (Figures 3(a) and 3(b)).

No differences in the percentages of B lymphocytes with bound platelets were found between the whole blood and PBMC analysis of HD (5.31 ± 0.54 vs. 6.06 ± 0.85, respectively) and SLE (7.6 ± 1.04 vs. 8.15 ± 1.29, respectively).

### 3.4. Phenotype of B Lymphocytes with Bound Platelets in HD and SLE Patients

Only in SLE patients, CD19+PLT+ lymphocytes had higher percentages of IgG+ and IgA+ cells than CD19+PLT- lymphocytes (Figures 3(c) and 3(d)). In HD and SLE patients, CD19+PLT+ lymphocytes had higher percentages of CD86+ cells and expressed higher levels of BAFFR than the counterpart CD19+PLT- lymphocytes (Figures 3(e) and 3(f)). However, IgM, CD38, and TACI expressions were comparable on CD19+PLT+ and CD19+PLT- lymphocytes from SLE patients and HD (data not shown).

### 3.5. IL-10 Production by B Lymphocytes with and without Bound Platelets

**Figure 4:**
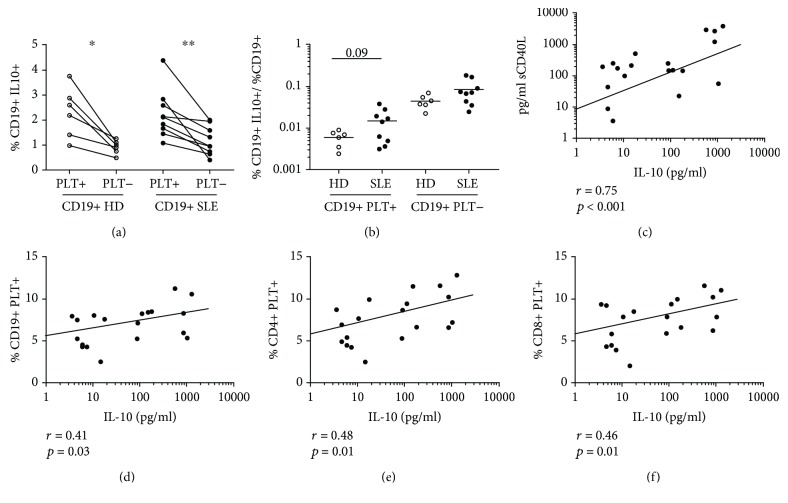
Association of B lymphocytes with bound platelets and IL-10 production and plasma levels. (a) Percentage of IL-10+ cells on CD19+PLT+ or PLT- and (b) percentage of CD19+PLT+ or PLT-IL-10+ according to the percentage of CD19+ producing IL-10 from HD (*n* = 6) and SLE (*n* = 9). (c) Correlation of plasma levels of sCD40L and IL-10 of 19 SLE patients. Correlation of plasma levels of IL-10 and (d) CD19+, (e) CD4+, and (f) CD8+PLT+ of 19 SLE patients. Two samples were not included because of the lack of plasma. Data are presented as the mean. Statistical analysis was the Wilcoxon test for paired samples and Mann-Whitney test for unpaired samples. Pearson's correlation was performed for correlation analysis. ^∗^*p* < 0.05 and ^∗∗^*p* < 0.01.

After 4 h of resting in medium, CD19+PLT+ lymphocytes contained higher percentages of IL-10+ cells than CD19+PLT- lymphocytes in HD and SLE patients (Figure 4(a)). In SLE patients, we observed a tendency to a higher percentage of CD19+PLT+IL10+ lymphocytes than in HD (Figure 4(b)). No differences in the percentage of IL-10+ cells were observed in CD19- lymphocytes in HD and SLE patients (data not shown).

We then analyzed the association of plasma IL-10 concentration with platelet activation, measured as sCD40L concentration, and with the percentage of circulating lymphocytes with bound platelets. In SLE patients, IL-10 concentration correlated with sCD40L levels and with the percentages of CD19+PLT+, CD4+PLT+, and CD8+PLT+ cells (Figures 4(c)–4(f)), but not in HD (*r* = 0.33, *p* = 0.41 for sCD40L; *r* = 0.58, *p* = 0.12 for CD19+PLT+; *r* = 0.52, *p* = 0.18 for CD4+PLT+, and *r* = 0.67, *p* = 0.067 for CD8+PLT+).

### 3.6. Relationship between Lymphocytes with Bound Platelets and Clinical Features

**Figure 5:**
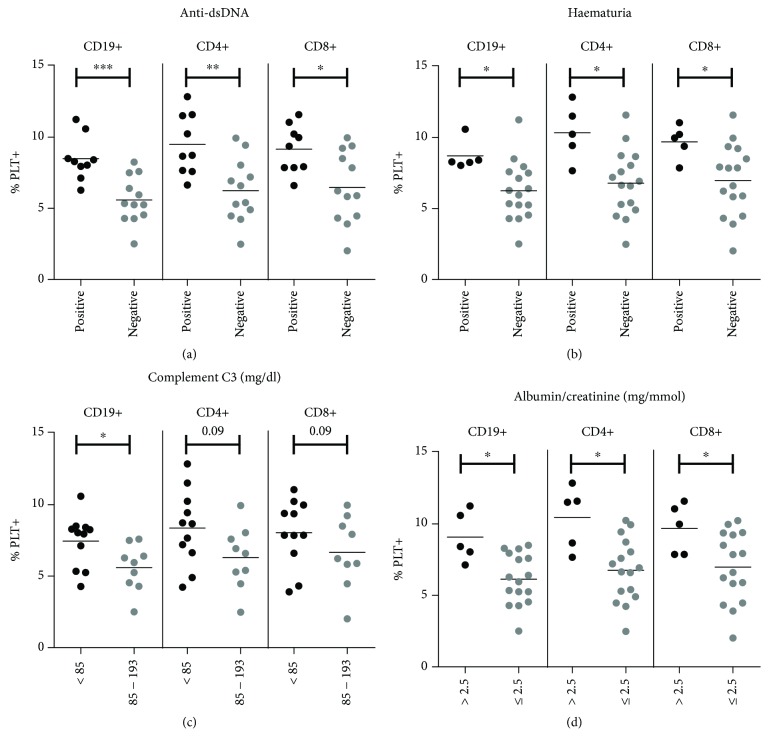
Percentage of lymphocytes with bound platelets in SLE patients according to clinical features. Whole blood from SLE patients (*n* = 21) was incubated with anti-CD4-PECy7, CD5-PE, CD19-PEDy647, and CD41a-FITC mAbs for flow cytometry analysis. Dot plots of percentages of CD19+ B lymphocytes and CD4+ and CD8+ T lymphocytes with bound platelets in SLE patients segregated according to (a) positive or negative anti-dsDNA, (b) positive or negative hematuria, (c) <85 or 85-193 mg/dl of C3, and (d) >2.5 or ≤2.5 mg/mmol of albumin/creatinine are shown. Data are presented as the mean. Statistical analysis was performed using the Mann-Whitney test. ^∗^*p* < 0.05, ^∗∗^*p* < 0.01, and ^∗∗∗^*p* < 0.001.

SLE patients with positive anti-dsDNA titers and those with hematuria had higher percentages of each subpopulation of lymphocytes with bound platelets than SLE patients with negative anti-dsDNA titers and without hematuria (Figures 5(a) and 5(b)). No correlation was found between the levels of anti-dsDNA antibodies and the percentage of CD19+PLT+. SLE patients with low complement C3 levels (<85 mg/dl) showed a higher percentage of CD19+PLT+ and a tendency to higher percentages of CD4+PLT+ and CD8+PLT+ than those with normal levels (85-193 mg/dl) (Figure 5(c)). SLE patients with albumin/creatinine levels > 2.5 mg/mmol showed higher percentages of CD19+PLT+, CD4+PLT+, and CD8+PLT+ than those with normal levels (≤2.5 mg/mmol) (Figure 5(d)). SLE patients with SLEDAI > 3 (patients with active disease suggestive of treatment change) [39, 40] showed higher percentages of CD19+PLT+, CD4+PLT+, and CD8+PLT+ than those with a SLEDAI ≤ 3 (CD19+PLT+: 8.51 ± 0.57 for >3 vs. 6.43 ± 0.49 for ≤3, *p* = 0.02; CD4+PLT+: 9.73 ± 0.66 for >3 vs. 7.11 ± 0.64 for ≤3, *p* = 0.02; and CD8+PLT+: 9.49 ± 0.59 for >3 vs. 7.22 ± 0.6 for ≤3, *p* = 0.03). Comparable percentages of each subpopulation of lymphocytes with bound platelets were observed when SLE patients were segregated according to medication and cutaneous or articular manifestations (data not shown).

## 4. Discussion

We found that the percentages of B lymphocytes with bound platelets were increased in SLE patients and correlated with plasma levels of sCD40L, IL-10, IgG, IgA, and IgM. B lymphocytes with bound platelets had upregulated activation markers and IL-10 production when compared to B lymphocytes without bound platelets. The highest percentages of lymphocytes with bound platelets were observed in SLE patients with positive anti-dsDNA and hematuria, a decreased complement C3, and an increased albumin/creatinine ratio. Our results suggest that the binding of platelets to lymphocytes could play a role in SLE disease through the modification of B cell function.

Joseph et al. described an increase in lymphocyte-platelet complexes and higher platelet activation in SLE patients [35]. However, these authors were unable to discriminate between different populations of lymphocytes. We have found that more B and T lymphocytes from SLE patients had bound platelets compared with HD and that these levels correlated with plasma sCD40L, a possible indirect measure of platelet activation [30, 31]. We have also found that T lymphocytes had more bound platelets than B lymphocytes in SLE patients suggesting a different interaction between platelets and T and B cells of SLE patients. Further experiments will reveal whether this increase is due to a particular T cell subset or a general increase of T cells with bound platelets in SLE. On the other hand, we did not analyze the activation state of free and bound platelets in these patients. It has been shown that the increased levels of plasma sCD40L indirectly indicate an increased platelet activation. However, CD40L is overexpressed on SLE T cells [28, 32, 33] and this molecule can be shed from the membrane of T lymphocytes [34], contributing to the soluble pool of sCD40L. On the other hand, using in vitro cultures, Danese et al. showed that platelets, but not T lymphocytes, from patients with inflammatory bowel diseases are the main source of the elevated sCD40L levels [41]. In addition, we also found in in vitro cocultures that sCD40L increased proportionally to the platelet-PBMC ratio [20]. An increase in platelet activation markers such as thromboxane [42], soluble and surface P-selectin and CD40L [43, 44], and PMPs [45] in the blood of SLE patients has been observed in previous studies. Since we found that platelet activation was associated with platelet binding to lymphocytes in SLE and other authors found that platelet activation is directly associated with SLE disease activity [28, 29], it is likely that platelet binding modifies lymphocyte function and, consequently, plays a role in SLE. We and others have shown that platelet bound preferably to memory T lymphocytes [19, 46]. Therefore, the described expansion of memory and effector T lymphocytes in SLE patients [47] could explain the increased binding of platelets to SLE than to HD lymphocytes. Several pairs of molecules, such as PSGL-1-P-selectin, have been shown to be involved in the lymphocyte-platelet complex formation in HD. However, we did not find any correlation between PSGL-1 and the percentage of platelet-bound lymphocytes in other inflammatory diseases (unpublished work). According to some reports, a modification of PSGL-1, the alpha3 fucosylation of the O-glycans, is crucial for P-selectin binding and it is restricted to polarized T cells [48, 49]. Therefore, it is likely that the analysis of fucosylated PSGL-1 could be more informative to understand the binding of platelets through P-selectin to SLE lymphocytes. In a different autoimmune context, we found that RA patients with the highest percentages of CD4+ cells with bound platelets had the lowest levels of IFN*γ* and IL-17 and the highest levels of plasma IL-10, with a less severe disease phenotype [19].

We found that the percentage of B lymphocytes with bound platelets and plasma sCD40L levels correlated with IgG and IgA levels and negatively correlated with IgM in SLE. It is well known that membrane CD40L, like sCD40L, is able to induce antibody production and signal B lymphocytes to switch IgG and IgA [22, 23, 26, 27]. With CD40L on the platelet surface, the binding of platelets to B lymphocytes [23] through CD40L-CD40 can induce the isotype switching. Accordingly, we found that B lymphocytes with bound platelets from SLE patients had higher membrane levels of IgG and IgA than B lymphocytes without bound platelets. An alternative explanation for our findings is that platelets bind preferentially to IgG+ and IgA+ B lymphocytes. Another intriguing result was that B lymphocytes without bound platelets from SLE patients had a higher expression of IgG and IgA than their counterparts from HD. This result could be related to the signal provided by the increased levels of sCD40L in the plasma of SLE patients.

Despite the new numbers of preswitched memory, we showed that more preswitched memory B lymphocytes had bound platelets in SLE patients than in HD. In addition, more preswitched memory B lymphocytes in SLE patients had bound platelets than in other B lymphocytes. Our results suggest that the preswitched memory stage of B lymphocytes favors platelet binding. Two previous observations can explain these results. First, we found that B lymphocytes with bound platelets had an increased expression of CD86 and BAFFR compared with B lymphocytes without bound platelets. Second, preswitched memory B lymphocytes in SLE expressed higher levels of the activation markers, CD80, CD95, and CD86, than in HD, as shown by Rodriguez-Bayona et al. The affinity of platelets for activated lymphocytes does not seem to be a mechanism limited to B lymphocytes since we, along with other authors, have shown that platelets also bind preferentially to activated T lymphocytes [18, 19], favoring their binding to endothelium to migrate [50, 51]. However, with our current experiments, we cannot conclude whether the binding of platelet to B lymphocytes upregulates the expression of activation markers or platelets bind preferably to B lymphocytes with an activated phenotype, such as preswitched memory. The addition of multiple activation markers for flow cytometry analysis will also reveal the full activated phenotype of the different subsets of B lymphocytes with bound platelets.

We showed that B lymphocytes with bound platelets had an increased percentage of IL-10-producing cells compared with B lymphocytes without bound platelets in SLE patients and HD. In addition, the plasma levels of IL-10 in SLE patients correlated with sCD40L plasma levels and the percentage of CD19+PLT+, CD4+PLT+, and CD8+PLT+. IL-10 has been shown to play a positive role in B lymphocyte survival, proliferation, switching, and autoantibody production [9], contributing to SLE pathology. Accordingly, plasma IL-10 levels in SLE patients are increased and correlate with SLEDAI and the production of anti-dsDNA antibodies [10]. In addition, increased IL-10 production was observed in lupus nephritis patients [52]. Our results suggest that increased platelet activation and, consequently, the higher number of B lymphocytes with bound platelets could induce the increased IL-10 production of B cells with a detrimental role in the SLE course. However, with our current experiments, we cannot conclude whether platelets bind to B lymphocytes producing IL-10 or whether the binding of platelets to B lymphocytes increases the IL-10 production.

The highest percentages of B and T lymphocytes with bound platelets were found in those SLE patients with active disease and those with renal manifestations. Further studies with a large cohort of patients and consistent data relating to the involvement of different organs will be required to find associations between platelet bound and lymphocytes with different organ manifestations. Since platelet binding is a consequence of platelet activation, the activation of platelets must be associated with the development of lupus nephritis. In experimental models, targeting platelets markedly reduced mortality, proteinuria, kidney histological score, and the levels of anti-DNA antibodies [53, 54]. In SLE patients, platelet activation, measured as sCD40L, is directly related to disease activity and renal manifestations [28, 29]. No differences in the percentages of B and T lymphocytes with bound platelets were found in SLE patients with cutaneous, articular manifestations or when they were segregated according to medication, even when we segregated SLE patients taking antiaggregating drugs such as aspirin or hydroxychloroquine. However, this fact was not surprising because only clopidogrel, but not aspirin, reduces the formation of leukocyte-platelet complexes throughout decreasing P-selectin expression on platelets [55]. However, we cannot discard that medication of SLE patients could affect lymphocytes with bound platelets. A study of lymphocyte-platelet complexes in a large cohort of patients before and after taking antiaggregating drugs or patients with and without antiphospholipids antibodies, which is known to be a potent platelet activator, will be required to study the modulation of these phenomena. We have demonstrated here that, in addition to the secretion of regulatory molecules, platelets can bind to lymphocytes in SLE. This binding is associated with an altered immune response, and these alterations are related to the clinical manifestations of SLE. However, one of our limitations is that we have excluded patients under a high dose of glucocorticoids (>10 mg/day) to avoid the influence of this treatment on our results. This exclusion could have biased the cohort to a less severe SLE, and it could explain the low frequency of patients with anti-dsDNA antibodies compared with other published cohorts. Another limitation is that lymphocyte-platelet complexes were analyzed at a one-time point in each patient. Further studies analyzing the same patient in a flare and in remission will be required to validate the potential of lymphocyte-platelet complexes as a tool to follow up SLE activity. With our current approach, we cannot discriminate if the increased percentage of B and T lymphocytes with bound platelets is involved in the development of SLE or if it is a consequence of the disease. Nevertheless, our findings suggest that controlling this binding is beneficial for the therapeutic regulation of autoimmunity.

## 5. Conclusions

Data in the current study show that SLE patients had a higher percentage of circulating lymphocytes with bound platelets than HD. This elevated percentage was associated with the plasmatic levels of Igs and IL-10 and sCD40L and B cell abnormalities described in SLE. In addition, SLE patients with suggestive renal manifestation and active disease had higher levels of lymphocytes with bound platelets. Taken all together, our findings suggest that the determination of the levels of circulating lymphocytes with bound platelets in SLE may prove to be a useful tool to follow up the activity of SLE disease and its renal manifestation. Moreover, controlling this binding may prove to be beneficial for the therapeutic regulation of autoimmunity.

## Figures and Tables

**Table 1:** Demographic, clinical, and laboratory characteristic data of study patients.

	SLE patients	HD
General conditions		
Age; year, mean ± SD	50.67 ± 12.58	49.06 ± 9.26
Gender (%) (*n*) women	100 (21)	75 (12)
Years of evolution, mean ± SD	12.41 ± 10.29	
SLEDAI; mean (range)	2.62 (0-12)	
Laboratory parameters		
ESR^a^ (mm/h, mean ± SD)	23.85 ± 20.02	
CRP^b^ (mg/l, mean ± SD)	2.63 ± 3.48	
Complement C3 (mg/dl, mean ± SD)	87.93 ± 20.86	
Complement C4 (mg/dl, mean ± SD)	14.12 ± 4.49	
Decreased C3 or C4 (%) (*n*)	80.95 (17)	
Anti-dsDNA (U/ml), mean (range)	74.8 (22-300)	
Positive anti-dsDNA (%) (*n*)	42.8 (9)	
Positive ANA (%) (*n*)	95.23 (20)	
Positive anti-Sm (%) (*n*)	4.7 (1)	
Positive antiphospholipid antibody (%) (*n*)	14.28 (3)	
Leukopenia and/or lymphopenia (%) (*n*)	14.28 (3)	
Clinical features		
Renal involvement (%) (*n*)	23.8 (5)	
Hematuria	23.8 (5)	
Albumin/creatinine > 2.5	23.8 (5)	
Cutaneous involvement (%) (*n*)	28.57 (6)	
Oral or nasal ulcers	4.7 (1)	
Alopecia	4.7 (1)	
Photosensitivity	23.8 (5)	
Butterfly erythema	9.5 (2)	
Arthritis (%) (*n*)	19.1 (4)	
Nervous system disorder	4.7 (1)	
Treatment (%) (*n*)		
None	9 (2)	
Mycophenolate	14 (3)	
Prednisone	48 (10)	
Hydroxychloroquine	62 (13)	
Azathioprine	24 (5)	
Tacrolimus	9 (2)	
NSAID^c^	14.28 (3)	
Aspirin	23.8 (5)	

^a^ESR: erythrocyte sedimentation rate. ^b^CRP: C-reactive protein. ^c^NSAID: nonstereoidal anti-inflammatory drugs.

## Data Availability

The data used to support the findings of this study are available from the corresponding author upon request.

## Abbreviations

CD: Cluster differentiation
dsDNA: Double-stranded DNA
HD: Healthy donors
IL: Interleukin
Ig: Immunoglobulin
mAbs: Monoclonal antibodies
PBMCs: Peripheral blood mononuclear cells
PLT: Platelets
PMP: Platelet-derived microparticles
SLE: Systemic lupus erythematosus
sCD40L: Soluble CD40L
SLEDAI: Systemic Lupus Erythematosus Disease Activity Index
TGF*β*: Transforming grow factor beta.
